# Case Report: Antibodies to the N-Methyl-D-Aspartate Receptor in a Patient With Multiple Sclerosis

**DOI:** 10.3389/fimmu.2021.664364

**Published:** 2021-04-23

**Authors:** Ran Zhou, Fei Jiang, Haobing Cai, Qiuming Zeng, Huan Yang

**Affiliations:** Department of Neurology, Xiangya Hospital, Central South University, Changsha, China

**Keywords:** anti-N-methyl-D-aspartate receptor antibody, multiple sclerosis, disease modifying treatment, case report, impaired consciousness

## Abstract

The association between multiple sclerosis and anti-N-Methyl-D-Aspartate receptor encephalitis is limited to merely a few case reports, and the exploration of the pathogenic mechanisms underlying the overlap of these two disease entities is very limited. Therefore, case reports and literature review on N-Methyl-D-aspartate receptor antibody in patients with multiple sclerosis are unusual and noteworthy. A young female had the first episode of paresthesia and motor symptoms with positive anti-N-Methyl-D-Aspartate receptor antibody and recovered after immunotherapy, and at the first relapse, the patient developed disorders of consciousness with positive anti-N-Methyl-D-Aspartate receptor antibody, findings of magnetic resonance imaging showed features of autoimmune encephalitis, which was also controlled by immunotherapy. At the second relapse, anti-N-Methyl-D-Aspartate receptor antibody turned negative while oligoclonal bands presented positive, and findings of magnetic resonance imaging showed features of multiple sclerosis. Afterwards, we followed the patient after receiving disease modifying treatment to monitor the efficacy and safety of teriflunomide. Based on literature review, demyelinating diseases patients with anti-neuronal antibody have complex, diverse and atypical symptoms; therefore, high attention and increased alertness are necessary for neurologists. Conclusively, anti-neuronal antibody may present in many neuroinflammatory conditions, and diagnostic criteria should be used with caution if the clinical presentation is atypical, and neurologists should not rely excessively on laboratory tests to diagnose neurological diseases. Timely and comprehensive examination and consideration as well as early standardized treatment are the key factors to reduce patient recurrence and obtain a good prognosis.

## Introduction

Multiple sclerosis (MS), as a chronic and predominantly immune-mediated disease of the central nervous system, is one of the most common causes of neurological disability in young adults globally (1). MS is characterized by the formation of lesions, inflammation, and the destruction of myelin sheaths of neurons, which results in diplopia, muscle weakness, and trouble with sensation or coordination (2). In clinical practice, the diagnosis of patients with MS is based on typical and common symptoms and signs of demyelinating disorders, while if atypical symptoms occur, the MS diagnosis should be reevaluated, with the help of long-term follow-up and multidimensional indicators, such as oligoclonal band (OCB) and evidence of spatial and temporal dissemination of the lesion on magnetic resonance imaging (3).

Anti-N-Methyl-D-Aspartate receptor (anti-NMDAR) encephalitis, characterized by the presence of IgG autoantibody against NMDAR in serum and cerebral spinal fluid (CSF), affects mainly pediatric and young adult female patients. The typical symptoms of anti-NMDAR encephalitis are psychosis, seizures, dyskinesias, memory deficits, speech problems, and autonomic instabilities (4). The finding of autoantibody against NMDAR in the central nervous system demyelinating diseases is limited to a few case reports, such as myelin oligodendrocyte glycoprotein (MOG) inflammatory demyelinating diseases (5), neuromyelitis optica spectrum disorders (NMOSDs) (6), and acute disseminated encephalomyelitis (ADEM) (7), and the association between anti-NMDAR antibody and MS is even more rare (8). Therefore, we reported a case report of a patient with MS who presented with a course of anti-NMDAR encephalitis. In addition, we followed the patient after receiving disease modifying treatment to monitor the efficacy and safety of teriflunomide. We present the following case in accordance with the CARE reporting checklist.

## Case Report

### Initial Admission (April, 2016)

**Figure 1:**
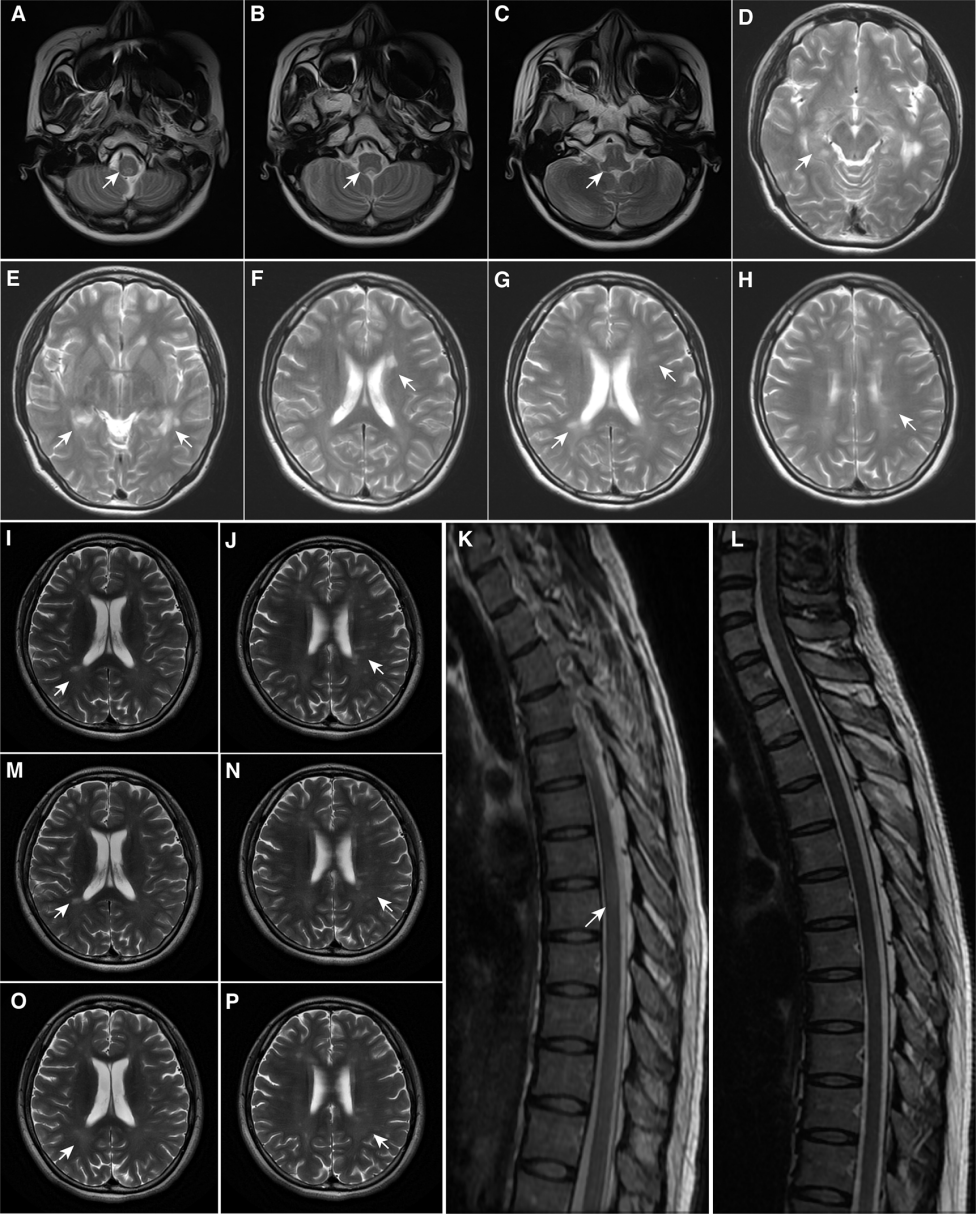
Magnetic Resonance Imaging Findings. **(A–C)** At first onset, MRI demonstrated patchy lesions in the medulla oblongata and dorsal pons; **(D–F)** At first recurrence, MRI demonstrated multiple lesions in the brain; **(G, H)** At second onset, MRI demonstrated pons lesions, consistent with the diagnosis of multiple sclerosis; **(I–K)** Before teriflunomide administration, MRI demonstrated new punctate enhancing lesions in the left frontal lobe and abnormal signals in the thoracic spinal cord at T6-7 of the skull and spinal cord; **(L–N)** After 6 months of teriflunomide use, MRI demonstrated that the lesions were reduced, especially in the spinal cord; **(O, P)** After 1 year of teriflunomide use, MRI demonstrated that the patient’s lesions were reduced. The arrowhead showing lesions in MRI. MRI, Magnetic resonance imaging.

A 16-year-old female undergraduate student from China suffered from headache and fever after catching a cold at the end of April 2016. Subsequently, she felt numbness on the soles of both feet and the numbness gradually elevated to her waist, accompanied by limb weakness and unstable walking. 10 days after the numbness, limb weakness, and unstable walking, the patient experienced diplopia, so she visited a hospital for treatment in May 2016. Physical examination revealed limited abduction of the left eye, limited adduction of the right eye, horizontal nystagmus, grade 4 muscle strength of four limbs, decreased position sense, deep tendon hyperreflexia, positive Hoffman and Babinski signs, positive Romberg sign, and waddling gait. Besides, the magnetic resonance imaging (MRI) test revealed that lesions in medulla oblongata and pontine tegmentum (**Figures 1A–C**), the findings of CSF disclosed positive anti-NMDAR antibody (**Table 1**), electroencephalogram showed mild abnormal electroencephalogram (slow basic rhythm), and the ultrasound examination revealed polycystic ovary. Although the patient had autoimmune encephalitis antibody and thus met the diagnostic criteria for anti-NMDAR encephalitis (9), the physician diagnosed her with a possible anti-NMDAR encephalitis, because of the patient’s atypical symptoms. After immunotherapy (Intravenous immunoglobulin, intravenous high-dose methylprednisolone, and oral glucocorticoid, the patient’s numbness, myodynamia, ataxia, and diplopia recovered, and anti-NMDAR antibody in the CSF turned negative. Since the patient had no obvious complaints and symptoms, the gynecologist recommended observation and follow-up for the polycystic ovary.

**Table 1:** Clinical characteristic laboratory data and treatments in first and relapse episode.

Characteristic	Initial Admission	Second Admission	Third Admission
**Symptoms**	Numbness and weakness of lower limbs, ataxia, diplopia;	Numbness and weakness of lower limbs, ataxia, diplopia, fatigue, and disorders of consciousness;	Fatigue, headache, decreased memory and numeracy;
**Laboratory Examinations**			
CSFP, mmH2O	165	108	140
CSF WBC*106/L	24	57	8
CSF anti-NMDAR-ab	Positive	Positive	Negative
Serum anti-NMDAR-ab	Negative	Negative	Negative
CSF antibodies to AMPA1, AMPA2, CASPR2, LGI1, GABAR	Negative	Negative	Negative
Serum antibodies to AMPA1, AMPA2, CASPR2, LGI1, GABAR	Negative	Negative	Negative
CSF oligoclonal bands	/	/	Positive
Serum oligoclonal bands	/	/	Negative
CSF/Serum AQP4-ab	/	/	Negative
CSF/Serum anti-MOG-ab	/	/	Negative
**Other Abnormal Findings**			
FT3	4.25, pmol/L (2.8-7.1)	1.99, pmol/L (2.8-7.1)	2.86, pg/ml (2.3-4.2)
FT4	14.18, pmol/L (12-22)	11.5, pmol/L (12-22)	1.13, ng/dl (0.89-1.76)
TSH	2.4, mIU/L (0.27-4.2)	0.405, mIU/L (0.27-4.2)	1.99, uIU/ml (0.55-4.78)
TGA	41.12, IU/mL (0-115)	268.9, IU/mL (0-115)	10.83, IU/ml (< 115)
TPO	5.02, IU/mL (0-34)	102.8, IU/mL (0-34)	12.87, IU/ml (< 34)
**Treatment of Acute Phase**	IVIg + Intravenous HDMP	IVIg + Intravenous HDMP	MMF (1.5 g/d)
**Treatment of Remission**	Oral GC (initial 60 mg/d and decreased 5 mg every 2 weeks)	Oral GC (initial 60 mg/d and decreased 5 mg every 4 weeks)	MMF (1.5 g/d) → Teriflunomide (14 mg/d)
		AZA (100 mg/d) → MMF (1g/d)	

IVIg, intravenous immunoglobulin; HDMP, high-dose methylprednisolone; AZA, azathioprine; MMF, mycophenolate mofetil; GC, glucocorticoid.

After discharge, the dose of oral glucocorticoid was gradually reduced and her condition had been stable until February 2017.

### Second Admission (February, 2017)

In February 2017, her symptoms recurred and she developed fatigue and disorders of consciousness. She was admitted to our institution. Physical examination revealed horizontal nystagmus in left eye, grade 4 muscle strength of four limbs, negative Babinski’s sign but positive Chaddock’s sign. The MRI examination showed multiple lesions and presented features of autoimmune encephalitis (**Figures 1D–F**) and the anti-NMDAR antibody in CSF turned positive again, as well as thyroid-associated antibody increased. Combined with her clinical symptoms, positive anti-NMDAR antibody and result of MRI examination, the patient met the diagnostic criteria for anti-NMDAR encephalitis. After immunotherapy (Intravenous immunoglobulin, intravenous high-dose methylprednisolone, and oral glucocorticoid), most symptoms improved, but mild fatigue and hypersomnia remained (**Table 1**).

After discharge, the dose of oral glucocorticoid was gradually reduced, but azathioprine was used to treat symptoms of hypersomnia and decreased memory and numeracy. However, due to severe impairment of liver function, azathioprine was discontinued and switched to mycophenolate mofetil.

### Third Admission (August, 2018)

In August 2018, she developed headache, fatigue, and severe decrease in memory and numeracy. Physical examination revealed limited adduction of the right eye, horizontal nystagmus, brisk tendon reflex, positive Hoffman and Babinski signs, and decreased pinprick sense over the upper limbs. The MRI test found that there were new lesions in the brainstem, periventricular, and corpus callosum (**Figures 1G, H**), the evoked potential test presented that the central segment of bilateral auditory pathway and the bilateral visual pathway were both injured, and OCB was positive in CSF. She was carefully evaluated for seizures, dyskinesia, psychiatric or neuropsychological symptoms, and we considered that her course and symptoms were most compatible with relapsing remitting MS (3), but the patient’s disturbance of consciousness and brain lesions at the first relapse might be closely related to anti-NMDAR antibody. Afterwards, the expanded disability status scale (EDSS) was used to evaluate the severity and progression of MS, and she reached 1.5. Neurologists advised the patient that teriflunomide (a disease modifying drug) should be used as soon as possible to control disease progression, but she adhered to mycophenolate mofetil due to financial burden (**Table 1**).

After discharge, the patient voluntarily stopped immunotherapy against medical advice in May 2019.

### Fourth Admission (August, 2019)

In August 2019, symptoms of decreased memory and numeracy relapsed and she developed urinary incontinence and narcolepsy, without significant abnormalities on physical examination. Her MRI test showed new spotted lesions in the left frontal lobe and a lesion in the thoracic spinal cord of thoracic spine 6-7 (**Figures 1I–K**) and her EDSS was 2.0. Subsequently, the patient was treated with immunotherapy (Intravenous high-dose methylprednisolone, oral glucocorticoid), and all symptoms were relieved after treatment. The patient decided to receive teriflunomide to decrease the disease activity.

## Diagnostic Assessment and Patient Perspective

We followed the patient closely since she received teriflunomide. On the one hand, to assess the safety of teriflunomide, we monitored blood routine and liver and kidney function before and 1, 3, 6, 12, and 18 months after teriflunomide use, and all parameters were within the normal range. In addition, a questionnaire was used to document her experience and adverse effects, and she felt that symptoms had improved significantly since the use of teriflunomide, with no other adverse effects other than mild alopecia.

On the other hand, to reflect the efficacy of teriflunomide, we evaluated her MRI tests, EDSS, and serological indicator before and after medication. As shown in (**Figures 1L–N**), spinal cord lesion volume was significantly reduced when patients used teriflunomide for half a year compared with that before teriflunomide use. In addition, **Figures 1O, P** show the lesions of the patient after 1 year of teriflunomide use, and a decrease in the volume and number of lesions was observed. Besides, after treatment with teriflunomide, her EDSS decreased from 2 to 1 and the level of serum Neurofilament light, a sensitive and clinically meaningful blood biomarker to monitor tissue damage and the effects of therapies in MS (10), experienced a significant drop from 66.66 pg/ml before treatment to 55.35 pg/ml at half a year, to 36.1 pg/ml at one year, to 27.72 pg/ml at one and a half years.

## Discussion

### Diagnostic Reasoning

On the first attack, although the anti-NMDAR antibody was positive, anti-NMDAR encephalitis presenting with pyramidal signs, sensory symptoms, ataxia and cranial nerve signs (ophthalmoplegia and nystagmus) with a brain stem inflammatory lesion would be extra-ordinarily unusual. Moreover, the patient’s symptoms were more consistent with the clinical features of inflammatory demyelinating disease. The absence of the examination was regrettable as the patient presented to other hospitals on her first admission. However, the positive OCB cannot be fully used as a basis for the diagnosis of MS, even if the patient has evidence of multispatial lesions and multitemporal lesions (positive OCB), because OCB can appear positive when anti-NMDAR antibodies are positive (11) (12). Conversely, if the OCB was negative at her first admission, the diagnosis of MS cannot be fully supported, and the diagnosis remains controversial due to positive anti-NMDAR antibodies. It is undeniable that a comprehensive examination is necessary to confirm the clinical diagnosis.

On the second attack, the patient’s MRI showing multiple intracranial lesions, especially in the hippocampus, were compatible with brain MRI characteristics of patients with anti-NMDAR encephalitis (13). Importantly, impaired consciousness was atypical of MS, while loss of consciousness, cognitive impairment and deficits in memory and numeracy are the most common symptoms of anti-NMDAR encephalitis (14), demonstrating that the re-positive anti-NMDAR antibody might play a pathogenic role in this episode and the patient met the diagnosis of anti-NMDAR encephalitis.

On the third and fourth attack, the patient’s MRI findings showed that the lesions were distributed in the paraventricular, medullary, cervical, and thoracic spinal cord segments, which were consistent with the MRI features of MS (15). Besides, the diagnosis of MS is supported by the patient’s symptoms as well as the positive OCB.

Overall, demyelination and antibody-associated encephalitis overlapped in this patient, and the differentiation between both clinical pictures is more a continuum then a dichotomic process.

### Diagnostic Challenges

The diagnosis of autoimmune encephalitis has always been a challenge. MRI, as an approach to aid in the diagnosis of autoimmune encephalitis, may not demonstrate specific abnormalities in most cases (16). For example, a case of anti- NMDAR encephalitis presented with MS-like demyelinated lesions (17). In terms of clinical features, anti-NMDAR encephalitis has diverse and complex clinical phenotype, including abnormal behavior (paranoia, hallucinations, and aggression), cognitive impairment, memory deficit, speech disorder, loss of consciousness, movement disorder, seizures, autonomic dysfunction. Importantly, it is common that some typical symptoms may be delayed, and the presence of anti-NMDAR antibody precedes the occurrence of anti-NMDAR encephalitis-related symptoms. Based on a retrospective analysis, there are 107 of 532 patients with anti-NMDAR encephalitis may not meet diagnostic criteria within the first month of symptom onset (14). Moreover, anti-NMDAR encephalitis may be comorbid with other diseases, resulting in atypical patient symptoms and diagnostic challenges, which requires long-time follow-up to retrospectively assess the disease course. Thus, detection of anti-NMDAR antibody in patient’s serum or CSF is considered the gold standard diagnostic test for anti-NMDAR encephalitis (18).

Actually, the maturation of anti-neuronal antibodies detection technology has brought new challenges. In our patient, the clinical symptoms presented at the first episode were not consistent with typical encephalitis, and the possibility and complexity of the disease could not be considered comprehensively if the disease was diagnosed based on laboratory tests alone. Therefore, at the first admission of patients, the incompleteness of examination is the deficiency of the diagnosis process, which is worth reflecting on. However, it is undeniable that this case reflects the current status of patient visits in the real world. In clinical practice, it is highly recommended that the diagnostic criteria should be used with caution and not used for over diagnosing neurological conditions based on a laboratory test, especially when the clinical presentation is atypical. In addition, the lack of comprehensive understanding of the diagnosis and clinical tests of the disease will result in treatment and diagnostic delays or even affect prognosis, especially when the standard treatment of anti-NMDAR encephalitis is useful for MS relapse, providing false reassurance of remission.

### Anti-NMDAR Encephalitis and Multiple Sclerosis

The positive anti-NMDAR antibody in demyelinating disorders are limited to a few reports (19), implying co-existence between demyelinating diseases and anti-NMDAR encephalitis (20). Undeniably, the differential diagnosis of multiple intracranial lesions is a challenging task, especially when the clinical features are neither specific nor unique, or laboratory tests do not correspond to clinical symptoms. We reviewed all case reports on MS and anti-NMDAR encephalitis, and the results of the literature review, containing the first case from England (21), along with one from Japan (22), two from Germany (8) (23), three from England (24) (25), one from Turkey (26), one from Austria (19), and one from China (27), are presented in **Table 2**. Through collation of previous cases, we found that only two male patient out of 10 had co-existence of anti-NMDAR encephalitis and MS, a proportion higher than the male-to-female ratio in MS (28) and anti-NMDAR encephalitis (29). In addition, the average age of these 10 patients was 30.1 years, and most cases presented with MS symptoms preceded by anti-NMDAR encephalitis, with the opposite course occurring in only two patients. Interestingly, our reported patient first presented with symptoms of inflammatory demyelinating disease of the central nervous system with positive anti-NMDAR antibodies, followed by anti-NMDAR encephalitis symptoms, and finally presented with MS. These 10 patients studied presented anti-NMDAR encephalitis symptoms, of which seizures, psychosis, and disorders of consciousness were more common. Similarly, most patients presented MS symptoms, such as limb weakness and numbness, diplopia, and optic neuritis. In terms of laboratory and MRI tests, most patients had positive OCB and anti-NMDAR antibody in serum or cerebrospinal fluid, and all MRI findings were more compatible with MS, although MS and anti-NMDAR encephalitis were not easily distinguishable on MRI (17). Unlike previous reports of anti-NMDAR encephalitis associated with ovarian teratomas (30), none of these 10 patients developed tumors. As far as treatment is concerned, immunotherapy has been the mainstream, with adjuvant symptomatic treatment, but there are differences in the treatment modalities given at different stages of the disease, such as the use of disease modifying drugs in the MS phase. The follow-up time of most cases reached half a year, while our follow-up time reached 18 months. In fact, it is necessary to observe and follow up patients with complex symptoms, which is conducive to understanding the changes in patients’ symptoms, especially the prognosis and changes in antibody titer levels so as to adjust medication.

**Table 2:** Previous case reports demonstrating the characteristics of patients with overlapping MS and anti-NMDAR encephalitis.

CASES	CASE1	CASE2	CASE3	CASE4	CASE5	CASE6	CASE7	CASE8	CASE9	CASE10	CASE11
**Year**	2010	2012	2014	2015	2015	2017	2017	2018	2020	2020	2021
**Country**	England	Japan	Germany	Germany	Austria	England	England	England	Turkey	China	China
**Author**	Johnston et al. (21)	Uzawa et al. (22)	Wachbisch et al. (23)	Fleishmann et al. (8)	Ramberger et al. (19)	Baheerathan et al. (24)	Baheerathan et al. (24)	Suleman et al. (25)	Gulec et al. (26)	Huang et al. (27)	Our case
**Age/sex**	32/F	33/F	33/M	33/F	23/F	32/F	29/M	41/F	26/F	19/F	16/F
**Course**	MS→NMDAR	MS→NMDAR	MS→NMDAR	MS→NMDAR	MS→NMDAR	NMDAR→MS	NMDAR→MS	MS→NMDAR	MS→NMDAR	MS→NMDAR	IDDs→ NMDAR→MS
**NMDAR Encephalitis symptoms**	Seizures, disorders of consciousness	Seizures, psychosis	Tonic spasms, paroxysmal tingling	Cognitive dysfunction, memory lost	Psychosis	Seizures, abnormal movements, and encephalopathy	Seizures, psychosis	Psychosis	Seizures, disorders of consciousness, agitation, hallucinations	Seizures, psychosis, sleep disorders	Disorders of consciousness, cognitive dysfunction
**Multiple Sclerosis symptoms**	Optic neuritis, right trigeminal sensory disturbance, dyskinesia	Optic neuritis, spinal cord and brainstem symptoms	Left hemiparesis	Paresthesia, reduced visual acuity, ataxia, dysphonia	Limb weakness, optic neuritis	Diplopia, facial weakness, and cerebellar ataxia	Limb weakness numbness	/	Limb weakness	Right eye visual loss and left limb paralysis	Limb weakness, diplopia
**CSF NMDAR antibody**	/	Positive	Negative	Positive	Positive	/	Positive	/	Negative	Positive	Positive
**Serum NMDAR antibody**	Positive	Negative	Positive	Positive	Positive	Positive	Positive	Positive	Positive	Positive	Negative
**OCB**	/	Positive	Positive	Positive	Positive	Negative	Positive	Positive	Positive	Positive	Positive
**MRI findings**	MS lesion beside lateral ventricle	Temporal lobe lesion, lateral ventricle MS lesion	MS lesion beside lateral ventricle with enhancement	Paraventricular MS lesions with enhancement, global brain atrophy, large paraventricular confluent lesions, brainstem and cervical spinal cord lesions.	MS lesion beside lateral ventricle	Demyelinating brain and spinal cord lesions	Demyelinating brain and spinal cord lesions	Demyelinating brain lesion	MS lesion beside lateral ventricle with enhancement	MS lesion beside lateral ventricle with enhancement	Multiple lesions in white matter
**Tumor**	No	No	No	No	No	No	No	No	No	No	No
**Treatment**	GC	GC	GC, rituximab	GC, PE, CP, natalizumab,	/	DMT	Improved spontaneously without immunotherapy	GC, IVIg, rituximab	Interferone-b1a, fingolimod, natalizumab, teriflunomide, PE, IVIg, HDMP, rituximab	IVIg, HDMP	HDMP, IVIg, PE, AZA, MMF, GC, teriflunomide
**NMDAR antibody evolution**	/	/	/	Decreased	Increased	Negative	Negative	/	Decreased	/	Negative
**Prognosis**	Recovery	Recovery	Recovery	Death	/	Free of relapses	/	Problems with short-term memory	Recovery	Recovery	Recovery
**Follow-up time**	/	6 months	12 months	6 months	/	10 months	/	/	12 months	/	18 months

OCB, oligoclonal band; IVIg, intravenous immunoglobulin; AZA, azathioprine; HDMP, high-dose methylprednisolone; MMF, mycophenolate mofetil; GC, glucocorticoid; PE, plasma exchange; DMT, disease modifying treatment; IDDs, inflammatory demyelinating diseases.

**Figure 2:**
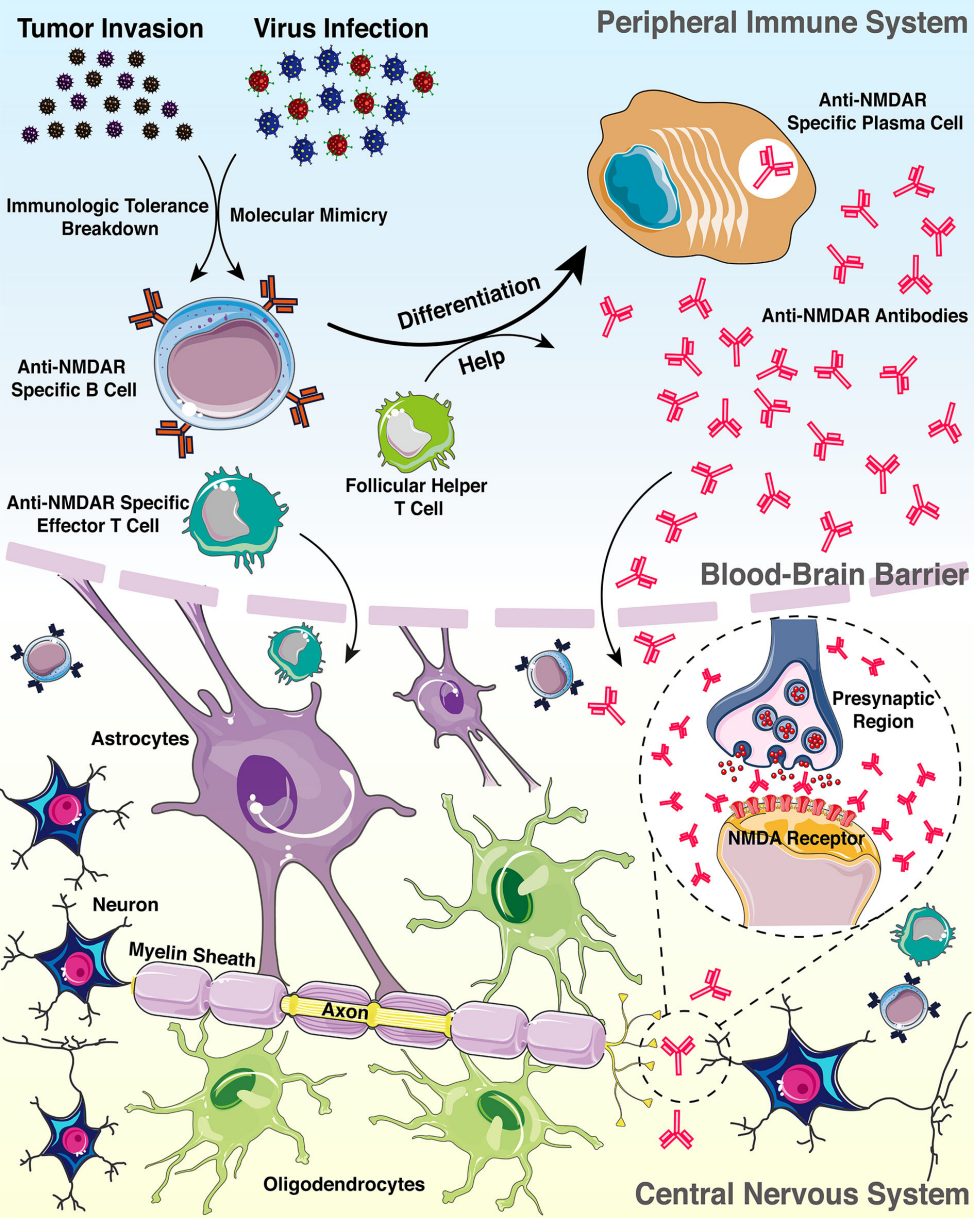
Pattern diagram of the possible mechanisms connecting anti-NMDAR antibody with demyelinating disorders.

To figure out whether the auto-antibodies indicate an evolving autoimmune process or represent a clinical non-significant epiphenomenon, we sought to explore the possible pathogenesis of anti-NMDAR antibodies in the inflammatory demyelinating disorders (**Figure 2**). Based on literature review, two confirmed triggers of anti-NMDAR antibody production are tumor (mostly ovarian teratomas) (31) and viral herpes infection (mostly simplex herpes infection) (32). Moreover, current findings indicated that 4%-7.5% of patients with anti-NMDAR encephalitis develop concurrent immune responses that may target not only glial antigens (The most frequent and clinically relevant glial antibodies are MOG and AQP4) but also neuronal surface antigens or receptors, and compared with patients with isolated anti-NMDAR antibody, those with coexisting glial antibodies were more likely to have a lower number of typical symptoms of anti-NMDAR encephalitis, and more frequent CSF pleocytosis, which is in accordance with the disease characteristics of our patient (33). In addition, previous studies have shown that NMDAR are present on the myelin sheath formed by oligodendrocytes, and a non-competitive NMDAR antagonist has successfully demonstrated to have a good clinical intervention effect on a variety of neurological diseases, including MS and spinal cord injury, which suggests that myelin injury resulting from a pathological anti-NMDAR antibody burst may activate or aggravate inflammation in MS (34). Therefore, in most patients, the NMDAR immune response drives the clinical picture, and specifically, due to the severe infection and immune derangements, NMDAR released by neuronal and myelin injury in the inflammatory environment leads to the disruption of immune tolerance (35), which may be the triggering mechanism of tissue injury and inflammatory response in MS. Although this patient did not show typical features of anti-NMDAR encephalitis at an early stage, it is undeniable that anti-NMDAR antibody play a role in the disease course of the patient, especially multiple lesions and disorders of consciousness at her first relapse (29).

In conclusion, neurologists should notice that anti-neuronal antibodies in demyelinating disorders can lead to atypical symptoms and ambiguous diagnosis, which might result in the inaccurate and untimely treatment of patients and affect the prognosis and quality of life of patients (36). Additionally, the molecular mimicry and the breakdown of immunologic tolerance toward NMDAR released following neuronal damage have been described as possible hypothesis for pathogenesis, but the mechanisms connecting anti-NMDAR antibody with demyelinating disorders remain to be further explored (27).

## Data Availability Statement

The original contributions presented in the study are included in the article/supplementary material. Further inquiries can be directed to the corresponding authors.

## Ethics Statement

Written informed consent was obtained from the individual(s) for the publication of any potentially identifiable images or data included in this article.

## Author Contributions

QZ and HY were responsible for providing study materials of the patient. RZ performed data analysis and interpretation. FJ helped with the analysis through constructive discussion. HC helped with the literature review. All authors contributed to manuscript writing and approved the submitted version.

## Funding

This study was supported by the grants from the National Natural Science Foundation of China (Grant Number: 81801203, 81771364) and the Natural Science Foundation of Hunan Province (Grant Number: 2019JJ50973).

## Conflict of Interest

The authors declare that the research was conducted in the absence of any commercial or financial relationships that could be construed as a potential conflict of interest.
